# Analysis and Validation of Contactless Time-Gated Interrogation Technique for Quartz Resonator Sensors

**DOI:** 10.3390/s17061264

**Published:** 2017-06-02

**Authors:** Marco Baù, Marco Ferrari, Vittorio Ferrari

**Affiliations:** 1 Department of Information Engineering, University of Brescia, Via Branze 38, Brescia 25123, Italy; marco.ferrari@unibs.it (M.F.); vittorio.ferrari@unibs.it (V.F.); 2 INO-CNR (National Research Council), Via Branze 45, Brescia 25123, Italy

**Keywords:** quartz crystal resonator, quartz crystal microbalance, contactless electromagnetic interrogation, resonant sensor, liquid solution microdroplet measurement

## Abstract

A technique for contactless electromagnetic interrogation of AT-cut quartz piezoelectric resonator sensors is proposed based on a primary coil electromagnetically air-coupled to a secondary coil connected to the electrodes of the resonator. The interrogation technique periodically switches between interleaved excitation and detection phases. During the excitation phase, the resonator is set into vibration by a driving voltage applied to the primary coil, whereas in the detection phase, the excitation signal is turned off and the transient decaying response of the resonator is sensed without contact by measuring the voltage induced back across the primary coil. This approach ensures that the readout frequency of the sensor signal is to a first order approximation independent of the interrogation distance between the primary and secondary coils. A detailed theoretical analysis of the interrogation principle based on a lumped-element equivalent circuit is presented. The analysis has been experimentally validated on a 4.432 MHz AT-cut quartz crystal resonator, demonstrating the accurate readout of the series resonant frequency and quality factor over an interrogation distance of up to 2 cm. As an application, the technique has been applied to the measurement of liquid microdroplets deposited on a 4.8 MHz AT-cut quartz crystal. More generally, the proposed technique can be exploited for the measurement of any physical or chemical quantities affecting the resonant response of quartz resonator sensors.

## 1. Introduction

AT-cut quartz crystal resonators (QCRs) are thickness-shear-mode (TSM) acoustic-wave resonators in which a thin quartz disk, obtained from a quartz rod sliced at an angle of 35.25° with respect to its optical axis, is sandwiched between two metal electrodes [1]. As a result of the piezoelectric nature of quartz, the application of an alternating electric field across the quartz disk produces a shear strain proportional to the electric potential. The QCR shows a set of resonant frequencies determined by the shear acoustic wave velocity and the crystal thickness.

These resonant frequencies are sensitive to a wide range of measurands, e.g., the mass deposited upon the crystal surface [2,3]. Based on this effect, quartz crystal microbalances (QCMs) are the QCRs commonly employed as mass sensors in gas phase, in vacuum, and in contact with liquids in many bio-analytic applications [4,5,6,7,8,9]. There are basically three different operation modes for QCR sensors. The first is based on an oscillator circuit in which the QCR is the element determining the frequency of oscillation. This method typically allows the measurement of the frequency variation due to different quantities, such as mass loading in QCMs, stress, or temperature, just to name a few. Oscillator circuits capable to provide output signals related to sensor frequency and energy dissipation for both the fundamental and the third harmonic have been reported [10]. The second operation mode involves impedance analysis allowing both the sensor frequency and energy dissipation data to be collected; also extending the analysis to multiple harmonics. Impedance analysis can be somewhat slow, and it can require expensive instrumentation, even if dedicated stand-alone interface circuits for the analysis of multiple-harmonic responses have been proposed [11,12,13]. The third operation mode is the Quartz Crystal Microbalance with Dissipation monitoring (QCM-D) [14] and it is based on the recording of the free decay of the QCM oscillations allowing simultaneous measurements of the sensor frequency, dissipation factor *D* and oscillation amplitude.

In all the above three cases, cabled links are required between the sensor unit and the readout electronic circuit or system. On the other hand, the possibility of contactless interrogation sensors can be attractive in applications where cabled solutions are not allowed, such as in closed volumes or packages. For instance, QCMs coated with suitable functionalizing polymers and operating as contactless gravimetric resonant sensors could be placed inside food packages for quality or spoilage monitoring. Alternatively, they could be adopted for the realization of smart sensing labels for sealed packages or drug conservation. Applications in liquid environments to monitor biological samples can be also viable. In contactless operation, the energy required to power the sensor unit needs to be made available on board. Battery-powered sensor units have been the most adopted solution, but they present the significant drawback of requiring periodical battery recharge or replacement. As an alternative, energy harvesting techniques can be adopted [15,16]. For applications involving hostile environments that may be incompatible with active electronics, an attractive solution is adopting passive sensors with energy supplied by an external interrogation unit. This is commonly done in the broad field of radio frequency identification (RFId) systems [17], as well as in remote sensing applications involving surface acoustic wave (SAW) sensors [18,19].

Because in the resonant measurement principle information is carried by the frequency of the readout signal, resonant sensing can be considered a robust approach in contactless operation to minimize the detrimental effect caused by the interrogation distance, which influences the readout signal amplitude. From this perspective, QCRs can be used as passive resonator sensors for the measurement of quantities affecting resonant frequency and/or quality factor. Techniques for contactless interrogation of quartz resonators have been previously studied, though they typically use special-electrode sensors or bare crystals [20,21]. Specifically, in [22] a technique based on measuring the admittance of a QCR sensor through an electromagnetic coupling between a primary coil and a secondary coil connected to the sensor was proposed. The reported analysis and results show that the variations of the readout QCR frequency depend on the mutual inductance between the coils and on their distance. This is a fundamental limitation in practical applications where keeping the distance fixed might be problematic or impossible.

The contactless electromagnetic principle proposed in this paper advantageously employs QCR crystals with ordinary electrodes. In addition, it grants a first order independence from the interrogation distance, since the mutual inductance between the coils acts only as a scaling factor on the signal amplitude.

In particular, the developed interrogation principle exploits the electromagnetic air coupling between two coils to perform a gated excitation of the resonator, followed by the sensing of the free transient response. The technique has been successfully employed with different piezoelectric sensors [23] and, with the aid of a static magnetic field, also with non-piezoelectric sensors, like silicon micro-electro-mechanical system (MEMS) resonators [24,25,26]. The technique also has a comparatively fast readout capability in the order of several readings per second, depending on the gating frequency, allowing the monitoring of rapid changes in the sensor parameters. To this purpose, dedicated post-readout techniques, based on autocorrelation analysis suitable for the implementation into embedded systems, have been developed for measuring the significant parameters of QCR sensors [27]. This contactless interrogation technique can simultaneously measure both the resonant frequency and quality factor (*Q* = 1/*D)* of the sensor, making it somewhat related to the QCM-D technique, but with the advantage of contactless operation.

This paper is dedicated to a theoretical analysis, based on the derivation of a lumped-element equivalent electromechanical circuit, and detailed investigation of the operating principle of the contactless interrogation technique. In particular, closed-form expressions for the frequency of the readout signal are presented for the first time, and the dependence on the equivalent model parameters are derived, supporting the analysis by numerical investigations. The theoretical predictions, in particular the independence of the readout signal frequency from the distance, are validated through experimental results on a developed system connected to AT-cut QCR sensors. In addition, the successful application of the proposed technique to measure the frequency shift due to microdroplets of water-sugar solution deposited on a quartz crystal resonator is reported.

## 2. Operating Principle

**Figure 1:**
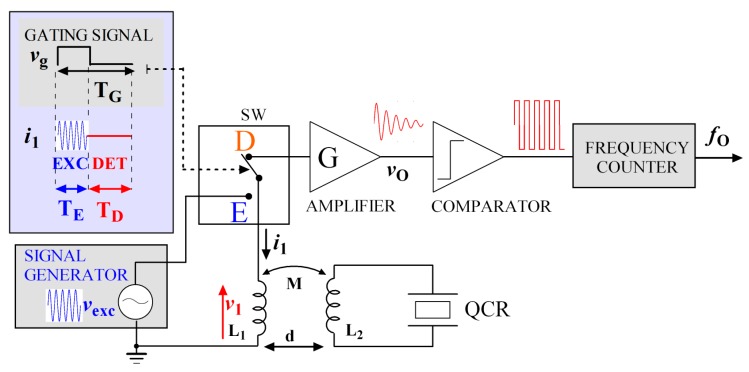
Simplified diagram of the contactless gated interrogation system.

Figure 1 illustrates the operating principle and block diagram of the proposed interrogation system for QCR sensors. The interrogation principle exploits the separation in time between driven excitation and free decay detection phases, somewhat similarly to what was previously proposed for silicon micromechanical resonators [24]. In the present case, however, the principle does not require magnets.

The developed interrogation system employs a primary coil with inductance *L*_1_ electromagnetically air-coupled to a secondary coil with inductance *L*_2_, connected to the electrodes of the QCR sensor. During the excitation phase a gating signal *v*_g_ sets the switch SW to the position E, connecting for a time interval *T*_E_ the primary coil *L*_1_ to the sinusoidal excitation signal *v*_exc_, which results in a gated sinusoidal signal at frequency *f*_exc_. By exploiting the electromagnetic air coupling between the two coils, the excitation signal is transmitted to the QCR which is excited into vibrations. The QCR is an electro-mechanical system which vibrates in thickness-shear mode and the TSM fundamental resonant frequency of its mechanical behavior will be indicated as *f*_r_ = *ω*_r_/2π. It can be noticed that, since the operating principle relies on the detection of the QCR free decaying response, ensuring that the excitation frequency *f*_exc_ is exactly equal to *f*_r_ is not strictly required, which is advantageous since *f*_r_ might not be exactly known in advance. Nevertheless, when *f*_exc_ approaches *f*_r_, the effectiveness of the excitation is increased and the amplitude of the detected signal rises.

In the detection phase, the gating signal *v*_g_ sets the switch SW to the position D for a time interval *T*_D_, disconnecting the excitation from the primary coil and, thus, also from the quartz resonator. It also connects the primary coil to the readout circuit. In this condition, the QCR undergoes decaying oscillations at frequency *f*_dr_, i.e., the mechanical damped resonant frequency. The initial amplitude of the oscillations is inversely related to the difference between *f*_exc_ and *f*_r_. Due to the piezoelectric properties of quartz, the mechanical vibrations of the resonator generate a current in the coil *L*_2_ and consequently an induced readout voltage *v*_1_ can be sensed back across *L*_1_.

The induced voltage *v*_1_ is further amplified by means of a high-impedance amplifier of gain *G* and then, by means of a zero-crossing detector, converted into an output square waveform with frequency *f*_o_ = *f*_dr_, which can be measured by a frequency counter.

### 2.1. Theoretical Analysis and Equivalent Model

Piezoelectric electroacoustic devices, such as AT-cut quartz resonators, can be studied by adopting the Mason distributed-parameter electromechanical circuit [3,28]. Around each resonant frequency corresponding to a given vibration mode, the Mason model can be simplified into the Butterworth-van Dyke (BVD) equivalent lumped-element circuit. The BVD circuit is composed of a motional, i.e., mechanical, branch and an electrical branch formed by the parallel capacitance *C*_0_. The motional branch comprises the series of inductance *L*_m_, capacitance *C*_m_, and resistance *R*_m_, which respectively represent the equivalent mass, compliance, and energy losses of the quartz crystal at the considered mode. In the following, the fundamental thickness-shear mode will be considered. With respect to the BVD circuit, the previously introduced mechanical resonant frequency *f*_r_ corresponds to the series resonant frequency of the motional branch, i.e., the frequency at which the reactance of the mechanical branch impedance vanishes, which can be expressed as *f*_r_ = 1/[2*π*(*L_m_C_m_*)^1/2^].

**Figure 2:**
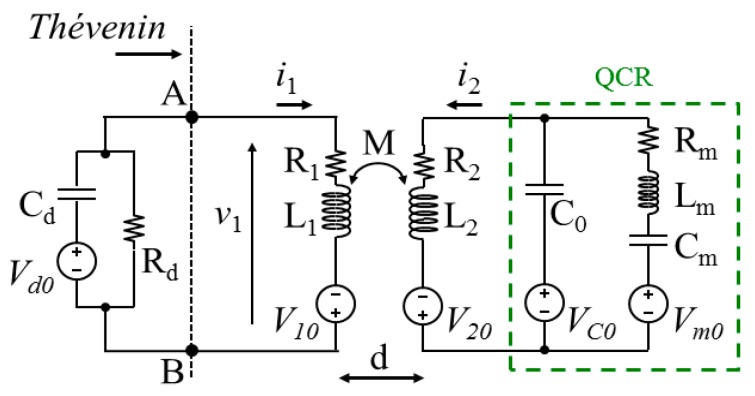
Lumped-element Butterworth-van Dyke (BVD) model of the quartz crystal resonator (QCR) sensor into the equivalent circuit of the interrogation system during the detection phase.

Accordingly, during the detection phase, the interrogation system can be modelled as illustrated in Figure 2. The two coils separated by a distance *d* are modelled by means of their mutual inductance *M* function of *d*, their equivalent series resistances *R*_1_, *R*_2_, and inductances *L*_1_, *L*_2_, i.e., the impedances of the primary and secondary coils are *Z*_1_ = *R*_1_ + *sL*_1_ and *Z*_2_ = *R*_2_ + *sL*_2_, respectively, where *s* is the complex frequency in the Laplace domain. The impedance *Z*_d_ = *R*_d_/(1 + *sR*_d_*C*_d_) represents the generic equivalent input impedance of the electronic detection circuit.

The voltage sources *V*_d0_ = *q*_Cd_(sC_d_)^−1^, *V*_10_ = *L*_1_*i*_L1_, *V*_20_ = *L*_2_*i*_L2_, *V*_C0_ = *qC*_0_(*sC*_0_)^−1^ and *V*_m0_ = *q*_Cm_(*sC*_m_)^−1^ − *L*_m_*i*_Lm_ represent the initial conditions of the electric capacitance *C*_d_ of the detection circuit input stage, of the primary and secondary coils *L*_1_ and *L*_2_, of the QCR electric capacitance *C*_0_ and of the QCR motional branch, respectively. The terms *q*_Cd_, *q*_C0_, and *q*_Cm_ are the initial charges on the capacitances *C*_d_, *C*_0_, and *C*_m_, respectively, and the terms *i*_L1_, *i*_L2_, and *i*_Lm_ are the initial currents in the inductances *L*_1_, *L*_2_, and *L*_m_, respectively, all of them taken at time *n*(*T*_D_ + *T*_E_) + *T*_E_, where *n* is an integer, i.e., at the beginning of the detection phase.

In order to derive an expression for the voltage *V*_1_ induced between nodes A and B in Figure 2, the Thévenin equivalent voltage source *V*_Th_ and impedance *Z*_Th_ of the right-hand part of the circuit can be expressed as:

(1a) $$V_{Th}=sMI_{2}-V_{10}$$

(1b) $$Z_{Th}=\frac{Z_{1}\left(Z_{2}+Z_{m}∥Z_{0}\right)-{\left(sM\right)}^{2}}{Z_{2}+Z_{m}∥Z_{0}}$$

where *Z*_m_ = *R*_m_ + *sL*_m_ + (*sC*_m_)^−1^ is the impedance of the QCR motional branch, *Z*_0_ = (*sC*_0_)^−1^ is the impedance of the capacitance *C*_0_, and *I*_2_ has the following expression:

(2) $$I_{2}=\frac{V_{20}(Z_{0}+Z_{m})+V_{C0}Z_{m}+V_{m0}Z_{0}}{Z_{2}Z_{m}+Z_{0}(Z_{2}+Z_{m})}$$

Hence the voltage *V*_1_ at the detection circuit input during the detection phase can be expressed by:

(3) $$V_{1}=V_{d0}\frac{sR_{d}C_{d}}{1+sR_{d}C_{d}}\frac{Z_{Th}}{Z_{Th}+Z_{d}}+V_{Th}\frac{Z_{d}}{Z_{Th}+Z_{d}}$$

In the limiting case of |*Z*_d_| → ∞, i.e., for high input impedance of the detection circuit, the readout voltage *V*_1_ becomes equal to *V*_Th_ and, hence, from Equation (1a), obtaining *V*_1_ reduces to the determination of the current *I*_2_.

Inserting the corresponding expression of each term in Equation (2) , *I*_2_ as a function of s can be put in the rational form *I*_2_(*s*) = *N*(*s*)/*D*(*s*) where *N*(*s*) and *D*(*s*) are given by:

(4a) $$N(s)=i_{L2}L_{m}C_{m}L_{2}C_{0}\left[s^{3}+\left(\frac{R_{m}}{L_{m}}+\frac{q_{C0}}{i_{L2}}\frac{1}{L_{2}C_{0}}\right)s^{2}+\left(\frac{C_{m}+C_{0}}{L_{m}C_{m}C_{o}}+\frac{q_{C0}}{i_{L2}}\frac{R_{m}}{L_{m}L_{2}C_{0}}\right)s+\frac{q_{C0}+q_{Cm}}{i_{L2}}\frac{1}{L_{m}C_{m}L_{2}C_{0}}\right]$$

(4b) $$D(s)=L_{m}C_{m}L_{2}C_{0}\left[s^{4}+\left(\frac{R_{2}}{L_{2}}+\frac{R_{m}}{L_{m}}\right)s^{3}+\left(\frac{1}{L_{m}C_{m}}+\frac{1}{L_{2}C_{0}}+\frac{1}{L_{m}C_{0}}\right)s^{2}+\left(\frac{R_{2}}{L_{m}C_{m}L_{2}}+\frac{R_{m}}{L_{m}L_{2}C_{0}}+\frac{R_{2}}{L_{m}L_{2}C_{0}}\right)s+\frac{1}{L_{m}C_{m}L_{2}C_{0}}\right]$$

From Equation (4a,b) it is possible in principle to derive a time expression for *i*_2_(*t*) and hence for *v*_1_(*t*) by making a partial fraction expansion of *I*_2_(*s*) and then taking the inverse Laplace transform of each term.

It must be remarked that, for the purposes of the present work, the main interest and specific goal is to determine the complex frequencies at which the electrical network composed of *R*_2_-*L*_2_-*C*_0_-*R*_m_-*L*_m_-*C*_m_ responds due to non-zero initial conditions on its reactive elements. On the other hand, determining the closed form expression of the time response *v*_1_(*t*) is unnecessary besides which it is very involved. These complex frequencies can be determined resolving *D*(*s*) = 0. The expression of *D*(*s*) is a fourth-order polynomial which can be factored in the product of two second-order polynomials as *D*(*s*) = *D*_0_(*s*^2^ + 2*α*_m_*s* + *ω*_m_^2^)(*s*^2^ + 2*α*_e_*s* + *ω*_e_^2^) where *D*_0_ is a normalization constant. As a consequence, it is expected that in the time domain *i*_2_(*t*) can be determined as the sum of two damped sinusoidal signals at the damped angular frequencies *ω*_dm_ = (*ω*_m_^2^ − *α*_m_^2^)^1/2^ and *ω*_de_ = (*ω*_e_^2^ − *α*_e_^2^)^1/2^ with natural angular frequencies *ω*_m_, *ω*_e_ and exponential decay times *τ*_m_ = 1/*α*_m_, *τ*_e_ = 1/*α*_e_, respectively. In addition, it can be observed that if *α*_m_ << *ω*_m_ and *α*_e_ << *ω*_e_, i.e., in the case of light damping, then *ω*_dm_ ≈ *ω*_m_ and *ω*_de_ ≈ *ω*_e_.

Solving the equation *D*(*s*) = 0, even if possible, is in general sufficiently involved to suggest adopting an approximate approach based on defined conditions. In particular, it will be demonstrated in the following that under specific assumptions, *ω*_dm_ corresponds to the damped angular frequency of the series resonant subcircuit of the mechanical branch *R*_m_-*C*_m_-*L*_m_, while *ω*_de_ corresponds to the damped angular frequency of the electrical resonant subcircuit composed of *R*_2_-*L*_2_-*C*_0_. In the more general case, this is not true because the two subcircuits are indeed coupled.

As a first step, the circuit of Figure 2 can be analyzed considering *R*_2_ = 0 and *R*_m_ = 0, which is equivalent to an undamped system. With this assumption, Equation (4b) reduces to:

(5) $$D_{u}(s)=L_{m}C_{m}L_{2}C_{0}\left[s^{4}+\left(\frac{1}{L_{m}C_{m}}+\frac{1}{L_{2}C_{0}}+\frac{1}{L_{m}C_{0}}\right)s^{2}+\frac{1}{L_{m}C_{m}L_{2}C_{0}}\right]$$

In this circumstance *D_u_*(*s*) does not contain terms of odd degree and the equation *D_u_*(*s*) = 0 can be regarded as a quadratic equation in the variable *p* = *s*^2^, i.e., *p*^2^ + *bp* + *c* = 0, and directly solved. By inspection of Equation (5), it is clearly *b* > 0, *c* > 0 and also for the discriminant *Δ* it holds that *Δ*= *b*^2^ − 4*c* > 0. Thus, for the solutions of the quadratic equation, two real negative roots *p*_1_ and *p*_2_ are expected, and hence the corresponding values of s are: *s*_1+_,_1−_ = ±*p*_1_^1/2^ = ±j*ω*_mu_ and *s*_2+_,_2−_ = ±*p*_2_^1/2^ = ±j*ω*_eu_. Recalling that *ω*_r_ = 2*πf*_r_ = (*L*_m_*C*_m_)^−1/2^ and defining *ω*_e0_ = (*L*_2_*C*_0_)^−1/2^ and *δω* = (*L*_m_*C*_0_)^−1/2^, the values of *ω*_mu_ and *ω*_eu_, corresponding to the two natural angular frequencies of the circuit, can be derived as:

(6a) $$\omega_{mu}={\left[\frac{(\omega_{r}^{2}+\omega_{e0}^{2}+\delta \omega^{2})+\sqrt{{\left(\omega_{r}^{2}+\omega_{e0}^{2}+\delta \omega^{2}\right)}^{2}-4\omega_{r}^{2}\omega_{e0}^{2}}}{2}\right]}^{1/2}$$

(6b) $$\omega_{eu}={\left[\frac{(\omega_{r}^{2}+\omega_{e0}^{2}+\delta \omega^{2})-\sqrt{{\left(\omega_{r}^{2}+\omega_{e0}^{2}+\delta \omega^{2}\right)}^{2}-4\omega_{r}^{2}\omega_{e0}^{2}}}{2}\right]}^{1/2}$$

It can be observed that *ω*_r_ and *ω*_e0_ represent the resonant angular frequencies of the series *L*_m_-*C*_m_ mechanical subcircuit and the parallel *L*_2_-*C*_0_ electrical subcircuit, respectively. Then Equation (6a,b) show that, by interconnecting the two subcircuits, a cross coupling is introduced, and each natural frequency of the network in general depends on the resonant frequencies of both subcircuits.

Under the hypothesis that *ω*_r_*L*_2_ « 1/(*ω*_r_*C*_0_), meaning that at the frequency *ω*_r_ the impedance magnitude of *L*_2_ is smaller than that of *C*_0_, the exact expressions in Equation (6a,b) can be approximated by a Taylor series arrested to the first order, leading to:

(7a) $$\omega_{mu}\approx \omega_{r}\left(1-\frac{1}{2}\frac{L_{2}}{L_{m}}\right)$$

(7b) $$\omega_{eu}\approx \omega_{e0}\left(1+\frac{1}{2}\frac{L_{2}}{L_{m}}\right)$$

From Equation (7a,b) it can be observed that both *ω*_mu_ and *ω*_eu_ depend on *L*_2_ and *L*_m_ and if *L*_2_ « *L*_m_ the natural frequencies of the network tend to the resonant frequencies of the two subcircuits.

The effect of damping introduced by *R*_m_ and *R*_2_ will be taken into account by numerical analysis in the following subsection. It is expected that the damped angular frequencies *ω*_dm_ and *ω*_de_ will depend in the general case on all the parameters of the circuit. However, it will be shown that, for light damping and for the range of variation of the parameters considered in the present work, *ω*_dm_ and *ω*_de_ are well approximated by their undamped counterparts given by Equation (7a,b).

### 2.2. Numerical Analysis

In the following the solutions of *D*(*s*) = 0, where *D*(*s*) is the expression in Equation (4b), will be computed numerically considering the typical values reported in Section 3 for both the parameters of the QCR and the electrical parameters. For the cases under examination, the solutions will be two pairs of complex conjugated numbers which, adopting the notation previously introduced, can be expressed as *s*_e_ = −*α*_e_ + j*ω*_de_ and *s*_m_ = −*α*_m_ + j*ω*_dm_. Simulations as a function of selected parameters of the equivalent circuit have been carried out in Matlab to validate the electrical equivalent model derived in Section 2.1.

**Figure 3:**
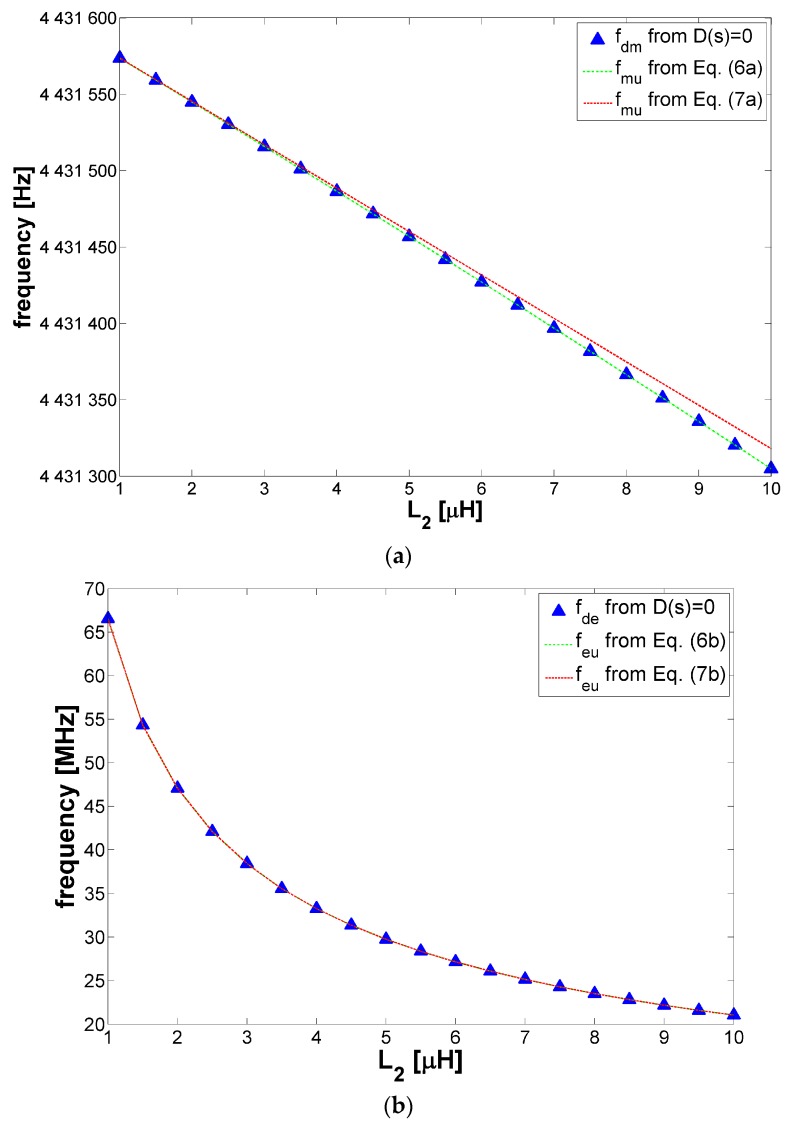
(**a**) Comparison of *f*_dm_ computed from Equation (4b) and the expressions of *f*_mu_ from Equations (6a) and (7a); (**b**) Comparison of *f*_de_ computed from Equation (4b) and the expressions of *f*_eu_ from Equations (6b) and (7b).

Figure 3a shows the comparison between the values of the damped mechanical frequency *f*_dm_ = *ω*_dm_/2*π* computed from Equation (4b) and the values of *f*_mu_ = *ω*_mu_/2*π* given by Equations (6a) and (7a). It can be observed that the numerical solution and the result of Equation (6a) are coincident while the results of Equation (7a) is within 3 ppm with respect to the numerical solution for *L*_2_ = 10 µH. It can be concluded that for the considered conditions, both the influence of the damping due to *R*_m_ and *R*_2_ and the presence of *L*_2_ do not affect the mechanical resonant frequency of the resonator and then *f*_dm_ ≈ *f*_mu_ ≈ *f*_r_.

Similarly, Figure 3b demonstrates that the electrical frequency *f*_de_ = *ω*_de_/2*π* computed from Equation (4b) and the expressions of *f*_eu_ = *ω*_eu_/2*π* from Equations (6b) and (7b) are also in remarkable agreement over the same range of variation of *L*_2_. Also in this case, it can be concluded that *f*_de_ ≈ *f*_eu_.

**Figure 4:**
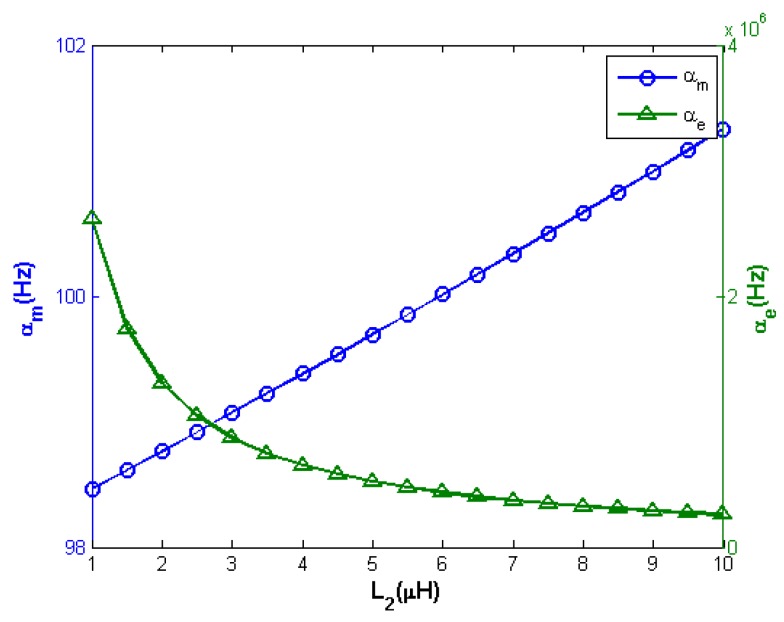
Attenuation constants *α*_m_ and *α*_e_ versus *L*_2_ computed from the numerical solution of *D*(*s*) = 0 as per Equation (4b).

Subsequently, for the same range of variation of *L*_2_, the attenuation constants *α*_m_ and *α*_e_ have been computed and the results are shown in Figure 4.

It can be seen that *α*_e_ is four orders of magnitude larger than *α*_m_, hence the contribution to *v*_1_ due to the electrical part falls off more rapidly than its mechanical counterpart and it is expected to quickly become negligible.

### 2.3. Additional Remarks

As reported in Section 2.1, the waveform of the current *i*_2_(*t*) is the sum of two damped sinusoidal signals at frequency *f*_dm_ and *f*_de_ with attenuation constants *α*_m_ and *α_e_*, respectively. In the numerical analysis reported in Section 2.2, it has been shown that *α*_e_ » *α*_m_. Thus, the damped sinusoidal signal at frequency *f*_de_ decays to zero much faster than the damped sinusoidal signal at frequency *f*_dm_, and, hence, the former can be neglected in the final expression of the waveform *i*_2_(*t*) which results:

(8) $$i_{2}(t)=I_{20}e^{-t/\tau_{m}}\cos(2\pi f_{dm}t+\theta_{2})$$

In Equation (8) the amplitude and phase coefficients *I*_20_ and *θ*_2_ are functions of both the initial conditions and the electrical and mechanical parameters of the system. The mechanical response of the QCR is read through the voltage *v*_1_, which in the time domain can be derived by taking the inverse Laplace transform of Equation (3) or, equivalently, multiplying *M* by the time derivative of Equation (8):

(9) $${v_{1}(t)=2\pi f_{m}MI_{20}e^{-t/\tau_{m}}\cos(2\pi f_{dm}t+\theta }_{m})-L_{1}i_{L1}\delta (t)$$

where the last additional term represents the contribution of the initial conditions on *L*_1_ and *θ*_m_ = *θ*_2_ – *π* − *arctan*(2*πf*_dm_/*α*_m_). From Equation (9) it can be seen that *v*_1_ is proportional to the natural frequency *f*_m_ and, notably, that the mutual inductance *M* acts only as a scaling factor for the amplitude of *v*_1_, without affecting the sensor response parameters *f*_dm_ and *τ*_m_. This is advantageous with respect to other contactless techniques like the one reported in [22] in which the resonant frequency of the QCR sensor is monitored by measuring the reflected admittance of the sensor through the primary and secondary coils. The significant limitation in this case is that the shape of the reflected admittance function versus frequency, and in turn the estimated QCR resonant frequency, depends on the mutual inductance between the coils and, as such, on their distance. Keeping the distance between the coils fixed is unpractical/unfeasible in most real applications. On the contrary, the gated technique here proposed which decouples excitation and detection is robust against the interrogation distance.

If the limiting case of *Z*_2_ → 0 is considered for a high quality factor resonator, i.e., the QCR is short-circuited during the detection phase and the mechanical damping is low, the voltage *v*_1_ is an exponentially lightly damped sinusoidal signal at exactly the frequency *f*_r_, i.e., the system detects the QCR oscillating at its series resonance.

## 3. Experimental Validation

**Figure 5:**
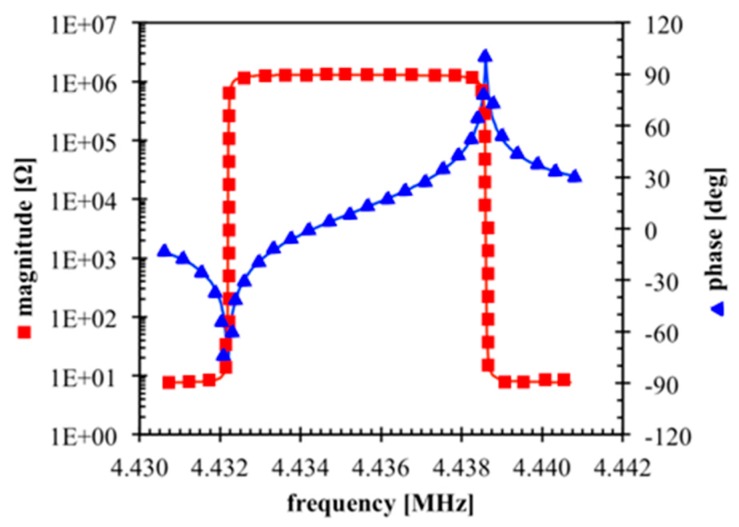
Measured impedance diagrams of the reference QCR.

The reference quartz resonator used to verify the derived theory and equivalent model of the contactless interrogation system is a 4.432MHz AT-cut 8-mm diameter crystal with 5-mm diameter gold electrodes. The impedance magnitude and phase diagrams of the sensor, measured with a HP4194A impedance analyzer, are shown in Figure 5. The extracted parameters of the BVD circuit are: *C*_0_ = 5.72 pF, *R*_m_ = 10.09 Ω, *L*_m_ = 77.98 mH, and *C*_m_ = 16.54 fF.

**Figure 6:**
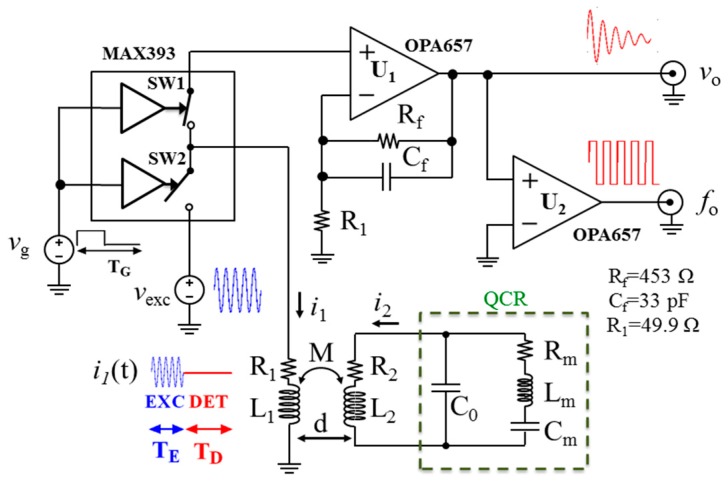
Schematic of the circuit for the time-gated interrogation of the QCR sensors.

Figure 6 shows the schematic diagram of the circuit realized to implement the contactless interrogation technique. The alternation between the excitation and detection phases is achieved by means of two pairs of normally-open (NO) and normally-closed (NC) electronic analog switches (SWs) (MAX393) driven by the gating signal *v*_g_(*t*). When the NC SW1 is open and the NO SW2 is closed, the coil *L*_1_ is connected to the excitation voltage *v*_exc_(*t*). Contrarily, when the NC SW1 is closed and the NO SW2 is open, the coil *L*_1_ is connected to the non-inverting amplifier with gain, corresponding to *G* in Figure 1, of 10 based on a wideband operational amplifier (OPA657) to obtain the output voltage *v*_o_(*t*). The output signal is subsequently squared by an additional operational amplifier used as a zero-crossing detector and its output is fed to a frequency counter to measure *f*_o_.

The QCR was driven with an excitation signal *v*_exc_ of frequency *f*_exc_ = 4.43 MHz and amplitude 5 V, whereas the gating signal *v*_g_ had a frequency of 175 Hz and duty cycle of 20%.

Planar spiral coils milled from copper-clad Flame Retardant (FR4) substrates of dimensions 3 cm × 3 cm were used for *L*_1_ and *L*_2_ during the tests. The coils were measured by means of a HP4194A impedance analyzer at 4.43 MHz. The primary and secondary coils had equivalent series inductance and resistance *L*_1_ = 8.45 µH and *R*_1_ = 5.07 Ω, and *L*_2_ = 8.53 µH and *R*_2_ = 5.22 Ω, respectively.

Optimal operation was achieved when the planes of coils were parallel and were aligned along their central out-of-plane axes of symmetry. In these conditions, the flux linkage between the coils and hence the mutual inductance are maximized. These operating conditions were always adopted in all the experimental results reported in the following. However, misalignments of a few millimeters and relative tilt of few degrees of the coils were found not to affect the interrogation process to a significant extent.

**Figure 7:**
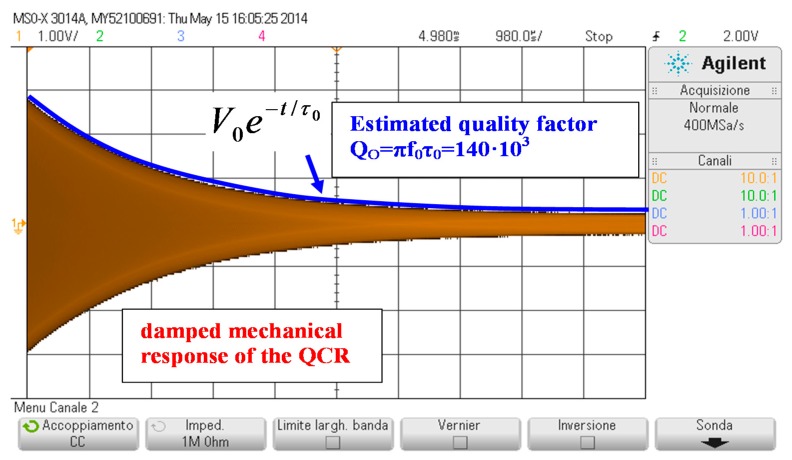
Measured readout voltage *v*_o_ versus time during the detection phase. From the exponential decaying envelope of the readout voltage, the quality factor of the electrically loaded quartz resonator sensor can be estimated.

Figure 7 shows a typical measured readout voltage *v*_o_ taken during the detection phase with the interrogation distance set to *d* = 5 mm. The interrogation system responds with an exponentially damped sinusoidal signal, as predicted by Equation (8). The readout frequency *f*_o_ was measured with a Fluke PM6681 frequency counter gated by the signal *v*_g_ and 30 repeated measurements in the same conditions showed a standard deviation of less than 1 Hz. The damped mechanical resonant frequency was measured to be *f*_dm_ = *f*_o_ = 4 431 871 Hz, while analyzing the decaying exponential envelope *V*_o_*exp*(−*t*/*τ*_o_) of the readout voltage *v*_o_ estimated the quality factor at *Q*_o_ = π*f*_o_*τ*_o_ = 140 × 10^3^. By substituting into Equation (7a) the values of the BVD parameters of the reference QCR, a frequency *f*_dm_ = 4 433 607 Hz was derived. Similarly, from the values of simulations of Figure 4, a quality factor *Q*_m_ = 142 × 10^3^ was estimated. Both values are in good agreement with the measured values, thus confirming the validity of the developed equivalent model.

In order to validate the proposed equivalent model, the behavior of the system has been tested for different values of the interrogation distance *d* and inductance of the primary and secondary coils *L*_1_ and *L*_2_.

**Figure 8:**
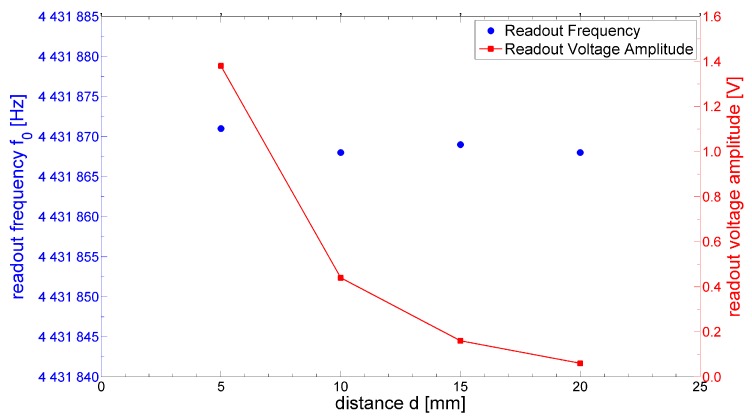
Measured readout frequency *f*_o_ and voltage amplitude for different values of the interrogation distance *d*.

Figure 8 shows the measured readout frequency *f*_o_ for different values of the interrogation distance *d*. It can be seen that the variations of *f*_o_ as the interrogation distance changes do not exceed 3 Hz. On the contrary, the amplitude of the readout voltage taken at a prescribed time *t*_0_ = 100 µs elapsed after the end of gated excitation decreases as the interrogation distance increases. This behavior confirms the prediction of the theoretical model and Equation (9), according to which the distance *d* changes the mutual inductance *M*, which in turns acts as a scaling factor on the amplitude of the readout voltage without, however, affecting the measured readout frequency.

**Figure 9:**
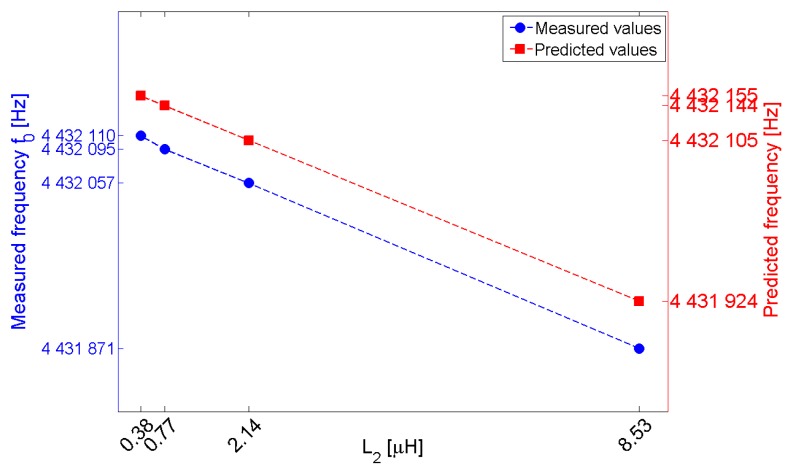
Measured readout frequency *f*_o_ and predicted frequency for different values of the inductance *L*_2_ of the secondary coil.

Figure 9 shows the measured readout frequency *f*_o_ for different values of the inductance *L*_2_ of the secondary coil while keeping *L*_1_ = 8.45 µH. The interrogation distance *d* is set to 5 mm. According to the model and to Equation (7a), the measured frequency is linearly dependent on *L*_2_ and, as *L*_2_ approaches zero, the readout frequency *f*_o_ approaches the QCR sensor series resonant frequency *f*_r_ = *f*_m_. The frequency offset between the measured and expected frequencies may well be caused by residual inaccuracies in the system parameter values used in the model.

**Figure 10:**
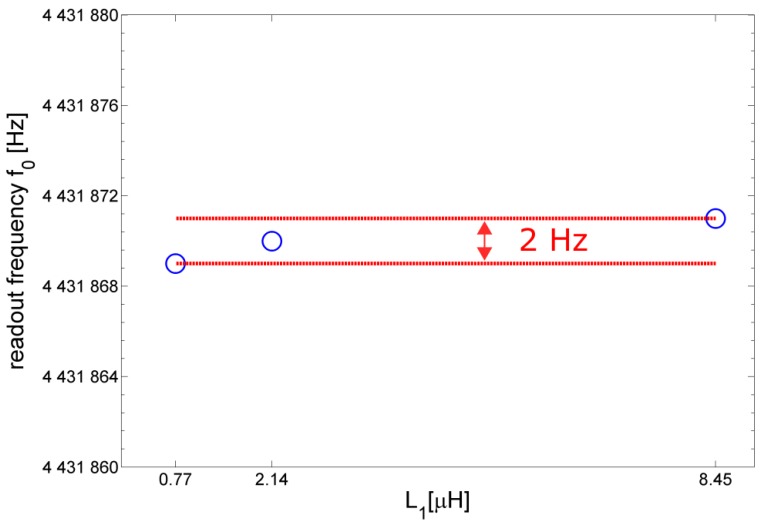
Measured readout frequency *f*_o_ for different values of the inductance *L*_1_ of the primary coil.

Figure 10 reports the measured readout frequency *f*_o_ for different values of the inductance *L*_1_ of the primary coil. It can be noticed that, as expected, the influence of *L*_1_ on *f*_o_ is negligible. However, decreasing the value of *L*_1_ worsens the signal-to-noise ratio (SNR), thus, lowering the repeatability of the readout frequency measurements. Therefore, it is desirable to choose a primary coil with a sufficiently high inductance *L*_1_ in order to grant an adequate SNR and repeatability in the contactless measurement operation.

## 4. Liquid Solution Microdroplet Measurements

The interrogation principle has been validated by detecting the frequency variations due to the deposition of microdroplets of liquid solution of sugar in water on a 4.8 MHz AT-cut quartz crystal.

**Figure 11:**
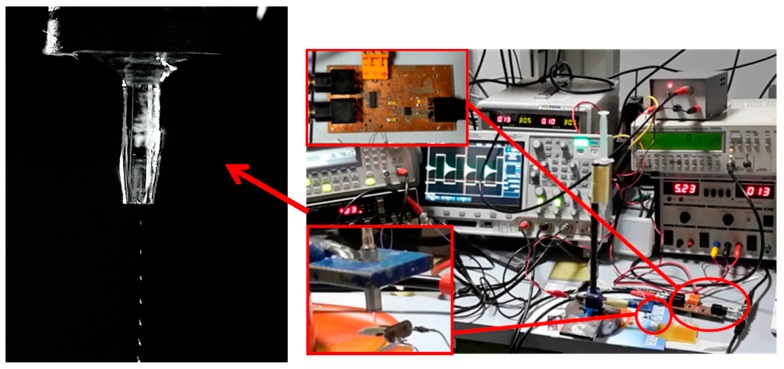
Experimental setup for the liquid solution microdroplet measurements. On the left a detailed view of the microdispenser.

A piezoelectric microdispenser (Microfab MJ-AB) with a 50 µm diameter orifice was adopted to deposit microdroplets on the sensor surface. For each deposition run *Dn*, the driving signal of the microdispenser was composed of *N* = 500 pulses at a driving frequency of 80 Hz, in order to obtain *N* microdroplets. The estimated volume of a single microdroplet was *v*_drop_ = 36 pl [10,11], obtaining a total volume of *V*_dep_ = *Nv*_drop_ = 18 nl per deposition run. A test solution of sugar in water at a concentration of *c* = 0.25 wt % was prepared for the measurements. Assuming that the density of the solution was about 1 g·cm^−3^, it results that the mass of sugar for each deposition was about *m*_dep_ = 45 ng. Figure 11 shows a picture of the experimental setup with a detailed view of the microdispenser ejecting a sequence of microdroplets. In the experimental activity, all the measurements were taken with the coils aligned and set at an interrogation distance *d* of about 5 mm.

**Figure 12:**
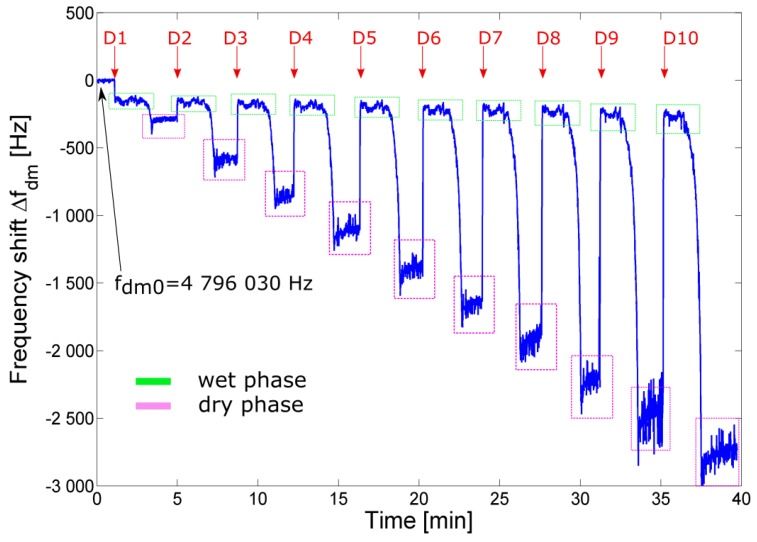
Frequency shift Δ*f*_dm_ of the output signal *v*_o_ relative to a sequence of consecutive 500-droplet depositions *D*_n_ of solution of sugar in water (*c* = 0.25 wt %).

Figure 12 shows the output frequency shift *Δf*_dm_ as a function of time during 10 deposition runs of the test solution. Each deposition was triggered about every 4 min. The unloaded damped frequency *f*_dm0_ of the quartz was 4 798 030 Hz. After each deposition run *D_n_*, the QCR sensor response was divided in two different phases termed in the following discussion as the *wet phase* and the *dry phase*. The *wet phase* corresponds to the situation where a fixed amount of volume *V*_dep_ of solution has been deposited and an initial steady-state frequency shift *Δf*_wet_(*n*) is measured before the evaporation process. The *dry phase* starts at the end of the evaporation process of water and the steady-state frequency shift |*Δf*_dry_(*n*)*|* is then measured. More specifically, it can be observed that at the moment of the first deposition *D*_1_ (at about *t* = 1 min) an initial frequency downshift is present. This is ascribed to the acoustic load given by both the liquid-solution density and viscosity. After the deposition, during the drying process, a second frequency downshift is observed (at about *t* = 3.5 min) which is ascribed to the transition of the acoustic load from *wet* to *dry* phases. The steady state (magenta boxes) given by the mass of the residual thin film of sugar, is then used to estimate *Δf*_dry_. In the *dry phase* it can be reasonably assumed that a sugar film of mass *m*_film_ = *n·m*_dep_ is formed on the surface of the QCR sensor. Starting from *D*_2_, an initial frequency upshift is present, suggesting that each fixed-volume added deposition dissolves the existing sugar film on the sensor (except for the first deposition *D*_1_) and essentially increases the sugar concentration in the deposited liquid solution. Each new deposition *D*_n_ increments the concentration of sugar solution *c*_s_ to *c*_s_ = *n × c* and causes a variation of the acoustic properties of the deposited liquid solution that is detected. After the deposition, where an initial steady-state (green boxes) is observed, the drying process starts and a frequency downshift is observed due to the transition from wet to dry phases. As a consequence, considering the (*n* + 1) deposition run, a frequency upshift is observed, such that |*Δf*_dry_(*n*)*| >* |*Δf*_wet_(*n +* 1)*|.* This behavior can be explained observing that in the wet phase *Δf_wet_*(*n*) is related to the concentration *c*_s_ of the solution, while in the dry phase *Δf_dry_*(*n*) is related to the mass *m*_s_ of sugar.

**Figure 13:**
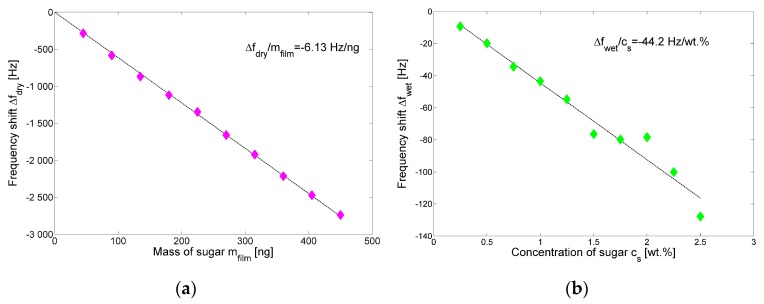
(**a**) Steady-state frequency shift Δ*f*_dry_ as a function of the mass of sugar *m*_film_ deposited (*dry phase*); (**b**) Steady-state frequency shift Δ*f*_wet_ as a function of sugar concentration *c*_s_ (*wet phase*).

Figure 13a shows the steady-state frequency shift *Δf*_dry_ derived from Figure 12 as a function of the deposited sugar mass *m*_film_, where the linear behavior obtained validates the operation of the QCR sensor in a gravimetric regime in the explored range with a sensitivity of about −6.13 Hz/ng. Figure 13b shows the steady-state frequency shift *Δf_wet_* derived from Figure 12 as a function of the sugar concentration *c*_s_, where a linear trend was obtained in the considered concentration range with a sensitivity of about −44.2 Hz/wt %.

In principle, the quality factor Q could be measured in the reported experiments, though they were not performed in this case with the adopted setup. A new contactless system which includes a tailored circuit performing heterodyne demodulation (frequency down-mixing) during the detection phase and autocorrelation analysis is reported in [27]. The new system allows frequency and quality factor measurements with an update rate of up to five measurements per second.

## 5. Conclusions

The present paper has addressed the theoretical study of a contactless interrogation system for quartz crystal resonator sensors which exploits the electromagnetic air coupling between two coils to perform a gated excitation of the resonator, followed by the sensing of the free transient response. The developed analytical model and related lumped-element equivalent circuit have shown that the proposed technique offers independence from the interrogation distance, which is advantageous with respect to other techniques requiring a fixed or known distance between the sensor and the interrogation unit. The predicted behavior has been investigated and validated experimentally. In addition, the technique has been applied to the measurement of deposition of microdroplets of a sugar-water solution. The proposed technique successfully measured relevant parameters of solutions in terms of solute and solvent. By adopting suitable elaboration techniques, such as correlation algorithms [27,29], improvements of the signal-to-noise ratio can be achieved. Future developments include the possibility of applying the technique to measurements on biological samples in closed volumes with proximate interrogation from outside.
